# Cell Autofluorescence and Lipofuscin

**DOI:** 10.3389/fmed.2015.00006

**Published:** 2015-02-19

**Authors:** Giovanni Di Guardo

**Affiliations:** ^1^Faculty of Veterinary Medicine, University of Teramo, Teramo, Italy

**Keywords:** autofluorescence, lipofuscin, lipofuscin-like substances, stem cells, epithelial cancer stem cells, cancer stem cells

I was greatly impressed by an article recently published in *Nature Methods* (1), in which intracellular autofluorescence was characterized as a biomarker for epithelial cancer stem cells (CSCs). Interestingly, this epithelial CSC-specific autofluorescence was also associated with a highly invasive and chemoresistant phenotype. Riboflavin (vitamin B_2_) – an ABCG2-selectively transported substrate – was identified, after autophagy had been ruled out, as the source of cell autofluorescence (1).

I have concerns about the fact lipofuscin was not included by the aforementioned authors among the putative causes of epithelial CSC autofluorescence. In this respect, it is worth mentioning that autofluorescent lipofuscin-like pigments have been reported to decrease, following differentiation, in murine embryonic stem cells (2). Furthermore, lipofuscin bodies, which are well-known markers for both aging and senescent cells, may be easily demonstrated, apart from autofluorescence, by means of Schmorl or Sudan Black histochemical stains (3). It should be additionally emphasized that lipofuscin bodies may occur in a variety of neoplasias, such as pancreatic tumors (4) and non-choroidal melanomas (5), with their number increasing in cell cultures from invasive mammary gland carcinomas (3).

As previously mentioned, autophagy was excluded as a possible source of epithelial CSC-specific autofluorescence (1). In this respect, it is also worth mentioning that the “macroautophagic system,” the best characterized among autophagy-related pathways (6), has been recently found to be hampered in aging by intracellular lipofuscin accumulation (7).

On the basis of what above, while the practical relevance and implications of the original findings by Dr. Miranda-Lorenzo and coworkers appear to be of crucial significance from the “fight against cancer viewpoint,” on one side, it should be also underscored, on the other side, that not including (or ruling out) lipofuscin and lipofuscin-like compounds among the factors responsible for epithelial CSC autofluorescence appears to be a (relevant) methodological error.

In conclusion, the potential contribution, if any, of lipofuscin and lipofuscin-like substances to the autofluorescent phenotype reported in epithelial CSCs (1) would deserve adequate consideration.

## Conflict of Interest Statement

The author declares that the research was conducted in the absence of any commercial or financial relationships that could be construed as a potential conflict of interest.
